# Machine Learning Prediction Models for Gestational Diabetes Mellitus: Meta-analysis

**DOI:** 10.2196/26634

**Published:** 2022-03-16

**Authors:** Zheqing Zhang, Luqian Yang, Wentao Han, Yaoyu Wu, Linhui Zhang, Chun Gao, Kui Jiang, Yun Liu, Huiqun Wu

**Affiliations:** 1 Department of Medical Informatics Medical School of Nantong University Nantong China; 2 Department of Information The First Affiliated Hospital Nanjing Medical University Nanjing China; 3 Department of Medical Informatics School of Biomedical Engineering and Informatics Nanjing Medical University Nanjing China

**Keywords:** digital health, gestational diabetes mellitus, machine learning, prediction model, prognostic model

## Abstract

**Background:**

Gestational diabetes mellitus (GDM) is a common endocrine metabolic disease, involving a carbohydrate intolerance of variable severity during pregnancy. The incidence of GDM-related complications and adverse pregnancy outcomes has declined, in part, due to early screening. Machine learning (ML) models are increasingly used to identify risk factors and enable the early prediction of GDM.

**Objective:**

The aim of this study was to perform a meta-analysis and comparison of published prognostic models for predicting the risk of GDM and identify predictors applicable to the models.

**Methods:**

Four reliable electronic databases were searched for studies that developed ML prediction models for GDM in the general population instead of among high-risk groups only. The novel Prediction Model Risk of Bias Assessment Tool (PROBAST) was used to assess the risk of bias of the ML models. The Meta-DiSc software program (version 1.4) was used to perform the meta-analysis and determination of heterogeneity. To limit the influence of heterogeneity, we also performed sensitivity analyses, a meta-regression, and subgroup analysis.

**Results:**

A total of 25 studies that included women older than 18 years without a history of vital disease were analyzed. The pooled area under the receiver operating characteristic curve (AUROC) for ML models predicting GDM was 0.8492; the pooled sensitivity was 0.69 (95% CI 0.68-0.69; *P*<.001; I^2^=99.6%) and the pooled specificity was 0.75 (95% CI 0.75-0.75; *P*<.001; I^2^=100%). As one of the most commonly employed ML methods, logistic regression achieved an overall pooled AUROC of 0.8151, while non–logistic regression models performed better, with an overall pooled AUROC of 0.8891. Additionally, maternal age, family history of diabetes, BMI, and fasting blood glucose were the four most commonly used features of models established by the various feature selection methods.

**Conclusions:**

Compared to current screening strategies, ML methods are attractive for predicting GDM. To expand their use, the importance of quality assessments and unified diagnostic criteria should be further emphasized.

## Introduction

According to the latest Global Diabetes Map (9th edition) released by the International Diabetes Federation, the number of patients with diabetes during pregnancy is increasing globally, with about 20.4 million (15.8%) women suffering from hyperglycemia; among them, 83.6% of cases were due to gestational diabetes mellitus (GDM) [1]. GDM, a common metabolic disease, is usually a transient disorder during pregnancy that resolves at delivery. Pregnant women with GDM are at greater risk of adverse pregnancy outcomes that threaten a normal birth. An oral glucose tolerance test (OGTT) is typically recommended to screen for GDM between the 24th and 28th weeks of gestation. Physicians usually measure the fasting plasma glucose concentration 1 to 2 hours after the patient ingests glucose [2]. The American Diabetes Association recommends that women be screened at the first prenatal examination to aid with the early identification of hyperglycemia risk. Nonetheless, GDM screening recommendations are controversial among international organizations regarding four aspects: (1) universal versus selective screening, (2) early pregnancy screening (ie, before pregnancy or at the first prenatal visit versus screening at 24-28 gestational weeks), (3) a one-step versus two-step approach, and (4) inconsistent diagnostic criteria (Table S1 in Multimedia Appendix 1) [3].

Machine learning (ML) methods have become favorable tools for disease prevention and management. For instance, the multivariate logistic regression (LR) model is a recognized ML algorithm for predicting diabetes and its complications. Furthermore, other methods, such as random forest (RF), extreme gradient boosting (XGBoost), and light gradient boosting machine (LightGBM), are also applied to diabetes-related problems. A growing number of studies have used such methods to identify risk factors of GDM and construct early prediction models for the disease [4,5]. ML presents a powerful tool for analyzing large amounts of diverse health care data and augmenting doctors’ capabilities. However, ML has limitations that can lead to inaccurate predictions in some clinical scenarios, and the significance of its assessment was highlighted in a real-world study [6]. The US Food and Drug Administration (FDA) has issued guidance on software as a medical device that explains risk stratification and the analytical and clinical validation required of artificial intelligence (AI) tools in health care. IDx-DR, the first FDA-approved ML application to help make screening decisions, achieved high sensitivity (87%) and specificity (91%) for diabetic retinopathy in primary care clinics [7]. Most of the published prognostic models for GDM also showed acceptable discrimination and calibration [8], but they vary in quality and perform inconsistently.

Few systematic analyses of ML models for GDM are currently available. Here, we conducted a thorough meta-analysis of the predictive value of ML in GDM using a quality evaluation by the Prediction Model Risk of Bias Assessment Tool (PROBAST) and compared ML models with universal and selective screening methods. Essentially, we wondered if ML could be a new GDM screening option.

## Methods

### Research Design

This study was conducted according to PRISMA (Preferred Reporting Items for Systematic Reviews and Meta-Analyses) guidelines (Table S2 in Multimedia Appendix 1) [9].

### Search Methods

The PubMed, Web of Science, IEEE Xplore, and China National Knowledge Infrastructure databases were searched for articles published in English or Chinese between August 2019 and October 2020. We built up the search strategy according to the PICO (population, intervention, control, and outcomes) principle; for our study, “P” represents GDM populations, “I” represents ML methods as interventions, “C” represents gold standards as controls, and “O” represents the outcomes of prediction and diagnosis, such as sensitivity, specificity, and accuracy (Table S3 in Multimedia Appendix 1). The details of the search keywords are listed in Textbox S1 in Multimedia Appendix 1. Additionally, the reference list of each identified study was manually searched to identify any additional studies. NoteExpress 3.2 (Aegean) [10] and EndNote X7 (Clarivate) [11] were employed to manage the studies and remove duplicate items.

### Inclusion and Exclusion Criteria

All studies included had to meet the following criteria: (1) published in English or Chinese; (2) included pregnant women from the general population, with a clear definition for GDM diagnosis; (3) included ML models for GDM prediction, with a clear description of the ML models; and (4) showed the performance of ML models, including sufficient data to enable the inference of sensitivity and specificity.

Articles in other languages, other types of articles (eg, reports and reviews), or those that used other measures for GDM detection were excluded. Four investigators (LY, WH, YW, and CG) participated in the literature screening to review all the studies that met the inclusion criteria. Each chosen article was screened at least twice, and disagreements were resolved by the reviewer (ZZ). Studies providing the most detailed information of variables and outcome indicators were kept for reference.

### Data Extraction

Data extraction was performed independently by two investigators (LY and LZ) according to the existing literature and the Transparent Reporting of a Multivariable Prediction Model for Individual Prognosis or Diagnosis (TRIPOD) standardized protocol [12]. A total of 25 studies were ultimately selected for the analysis. The following data were extracted from each study: (1) demographic information (ie, the country in which the data were gathered, the setting, the data source, the study design, the prediction temporality, and the outcome definition); (2) the data division method, the feature selection algorithms, the features of the model training, the ML prediction model type, and the model validation and application; (3) prediction outcomes, including accuracy, sensitivity, specificity, and area under the receiver operating characteristic curve (AUROC); and (4) funding and ethics approval.

### Quality and Bias Assessments

The PROBAST [13], which includes a total of 20 signaling questions in four domains (ie, participants, predictors, outcome, and analysis), was used as a tool for assessing the risk of bias and applicability of each included study.

### Statistical Analysis

The performance of each ML model was described using the primary outcome measures of discrimination and calibration. Model discrimination or concordance index (C-index) is similar to the AUROC [14] and indicates its diagnostic or prognostic discrimination ability as none (AUROC ≤0.6), poor (AUROC >0.6 to 0.7), fair (AUROC >0.7 to 0.8), good (AUROC >0.8 to 0.9), or optimum (AUROC >0.9 to 1). Model calibration is a metric of goodness of fit that assesses the agreement between observed and predicted outcomes and reflects the stability of the model via calibration plots. The diagnostic odds ratio (DOR) was also calculated via the following equation:

DOR = PLR / NLR **(1)**

where PLR is the positive likelihood ratio and NLR is the negative likelihood ratio. The PLR and NLR were calculated to express how frequently the model predicted GDM among the individuals with GDM versus among those without GDM using the following equations:

PLR = Sensitivity / (1 − Specificity) **(2)**

NLR = (1 – Sensitivity) / Specificity **(3)**

In this meta-analysis, the Meta-DiSc software program (version 1.4) [15] was used to calculate the pooled estimates of AUROC, sensitivity, specificity, PLR, NLR, and DOR. It was used to summarize the data from the included studies and graphically investigate the homogeneity among the studies. The I^2^ test was used to assess the statistical heterogeneity among the included studies. An I^2^ value of more than 75% indicated high heterogeneity among the studies [16]. The analysis of the included studies was divided into primary and subgroup analyses to judge the performances of the ML methods in predicting GDM in different clinical scenarios. Sensitivity analysis, subgroup analyses, and a meta-regression were also conducted to gain insight into potential sources of interstudy heterogeneity due to selector or inclusion criteria bias. The abilities of the different ML algorithms (eg, LR, Bayesian model, TreeNet, and GA-CatBoost [genetic algorithm category boosting]) for predicting GDM are discussed in the Subgroup Analysis section. The four predictive models with the highest and the lowest values were excluded from the sensitivity analysis to assess the impact of outliers on pooled sensitivity and specificity.

## Results

### Study Selection

**Figure 1:**
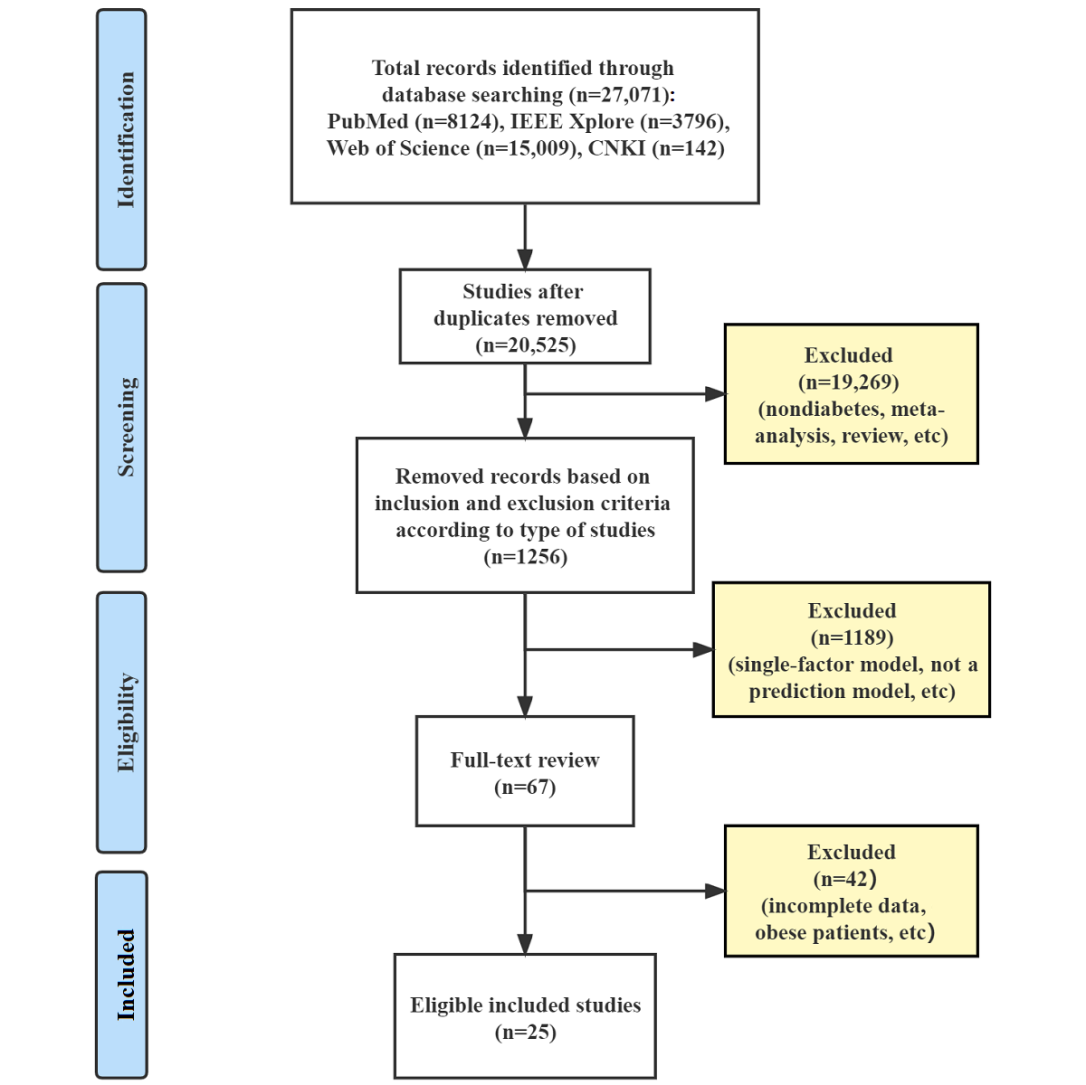
PRISMA (Preferred Reporting Items for Systematic Reviews and Meta-Analyses) flow diagram for study selection. CNKI: China National Knowledge Infrastructure.

A total of 27,071 studies were initially identified; of those, 1256 (4.6%) that discussed GDM were subjected to abstract screening. A total of 67 studies were subjected to full-text review; of those, 25 (37%) were included in the meta-analysis [17-33]. Figure 1 shows the PRISMA flow diagram of the study selection process.

### Study Characteristics

The articles’ years of publication ranged from 2004 to 2020; 10 out of 25 (40%) were published in 2020 (Figure S1 in Multimedia Appendix 1). All studies included women older than 18 years without a history of heart or cerebrovascular disease or vital organ dysfunction. Out of 25 studies, 9 (36%) included patients with a history of GDM, while other studies excluded those cases with a history of GDM (Tables S4-S6 in Multimedia Appendix 1). The source data for ML training were mostly obtained from medical centers and maternity hospitals; some also included self-administered questionnaires. Out of 25 studies, 9 (36%) were conducted using data from a population-based prospective cohort or multicenter study. The sample size of the included studies varied from 134 to 66,687 participants.

Feature selection is an important step for ML training. Xiong et al [19] developed a prediction model for GDM risk in the first 19 weeks of gestation with several hepatic, renal, and coagulation function measures; they observed that a cutoff of prothrombin time and activated partial thromboplastin time could reliably predict GDM with a sensitivity of 88.3%, a specificity of 99.47%, and an AUROC of 94.2%. Maternal age, family history of diabetes, BMI, and fasting blood glucose were the four most commonly used features of the established models, whereas pregnancy-associated plasma protein A, leptin, lipocalin-2, adiponectin, weight gain, and soft drink intake during pregnancy were used in only one or two models each. Table 1 [17-41] summarizes the most frequent features included in the prognostic models.

**Table 1:** The most frequent factors included in risk prediction models for gestational diabetes mellitus.

Study first author, year	Factors included in models												
	MA^a^ (n=19)	FHD^b^ (n=14)	BMI (n=12)		FPG^c^ (n=11)	PBMI^d^ (n=8)	HD^e^ (n=8)	Ethnicity (n=6)	TG^f^ (n=5)	HbA_1c_^g^ (n=4)	SBP^h^ (n=3)	Height (n=3)	hsCRP^i^ (n=3)
Gao, 2020 [21]	✓^j^	✓	✓								✓	✓	
Liu, 2020 [17]	✓			✓		✓							
Miao, 2020 [28]		✓		✓					✓				
Tan, 2020 [41]	✓	✓		✓		✓							
Wu, 2020 [18]	✓	✓		✓			✓						
Xiong, 2020 [19]													
Ye, 2020 [20]	✓		✓	✓		✓	✓		✓	✓			
Zhang, 2020 [39]	✓	✓		✓		✓	✓			✓	✓		✓
Snyder, 2020 [40]	✓					✓		✓					
Cui, 2019 [25]	✓	✓				✓							
Zheng, 2019 [24]	✓		✓	✓					✓				
Nombo, 2018 [26]		✓	✓										
Sweeting, 2018 [27]		✓	✓				✓	✓	✓				
Xiao, 2018 [38]	✓		✓	✓					✓	✓		✓	
Huang, 2017 [23]	✓	✓		✓		✓							
Wu, 2017 [22]	✓		✓	✓								✓	✓
Gabbay-Benziv, 2015 [30]	✓		✓				✓	✓			✓		
Thériault, 2015 [29]	✓	✓	✓				✓	✓		✓			✓
Eleftheriades, 2014 [31]	✓												
Pintaudi, 2013 [32]		✓		✓		✓							
Savona-Ventura, 2013 [33]	✓			✓									
Tran, 2013 [34]	✓		✓										
Teede, 2011 [35]	✓	✓					✓	✓					
Vanleeuwen, 2009 [36]		✓	✓				✓	✓					
Caliskan, 2004 [37]	✓	✓	✓										

^a^MA: maternal age.

^b^FHD: family history of diabetes.

^c^FPG: fasting plasma glucose.

^d^PBMI: prepregnancy BMI.

^e^HD: history of diabetes.

^f^TG: triglyceride.

^g^HbA_1c_: hemoglobin A_1c_.

^h^SBP: systolic blood pressure.

^i^hsCRP: high-sensitivity C-reaction protein.

^j^A checkmark (**✓**) indicates that the factor was included.

The LR model was the most universally used model in the 25 studies (n=17, 68%) for predicting GDM risk, while 5 (20%) studies assessed the performance of other ML methods (ie, GA-CatBoost, XGBoost, Bayesian model, TreeNet, gradient-boosting decision tree [GBDT], adaptive boosting [AdaBoost], LightGBM, Vote, and RF). For measuring deep learning performance, AUROC and the Youden index were most commonly used. AUROC was used in studies that did not provide the C-index. Out of 25 studies, 2 (8%) did not report metrics of model discrimination. Of the 25 studies, only 7 (28%) presented calibration measures. Internal validation was performed in 13 studies (52%) using random split or k-fold cross-validation and bootstrapping. Only 4 studies out of 25 (16%) performed external validation.

### Quality Assessment

Items from the PROBAST checklists (Multimedia Appendix 2) were used to assess the risk of bias and applicability of the prognostic prediction model studies. According to the criteria, the biases of participants in 4 out of 25 (16%) studies [30,31,33,36] were moderate, mainly due to debatable criteria, while biases in the other studies were low. Out of 25 studies, 24 (96%) study groups had a low bias of predictors, while 1 (4%) [32] had a moderate risk of bias because the prediction assessment was created with knowledge of the outcome data. The bias of outcome in 6 (24%) studies [22,30,33,35-37] was moderate due to the diagnostic criteria, while the others were low. A total of 8 (32%) groups had a moderate bias of analysis [21,23,27,31-33,36,37] and 1 (4%) [30] showed a high risk of bias due to an unreasonable number of participants with outcomes. The overall bias rating of 10 (40%) groups [21-23,27,30,32,33,35-37] was moderate. Overall concerns regarding the applicability rating of 7 (28%) studies [21-23,27,29,32,33] were moderate because of excessive features in models making it difficult to collect data in actual use, whereas others were low (Table S7 in Multimedia Appendix 1).

### Performance of ML Models for GDM Prediction

**Figure 2:**
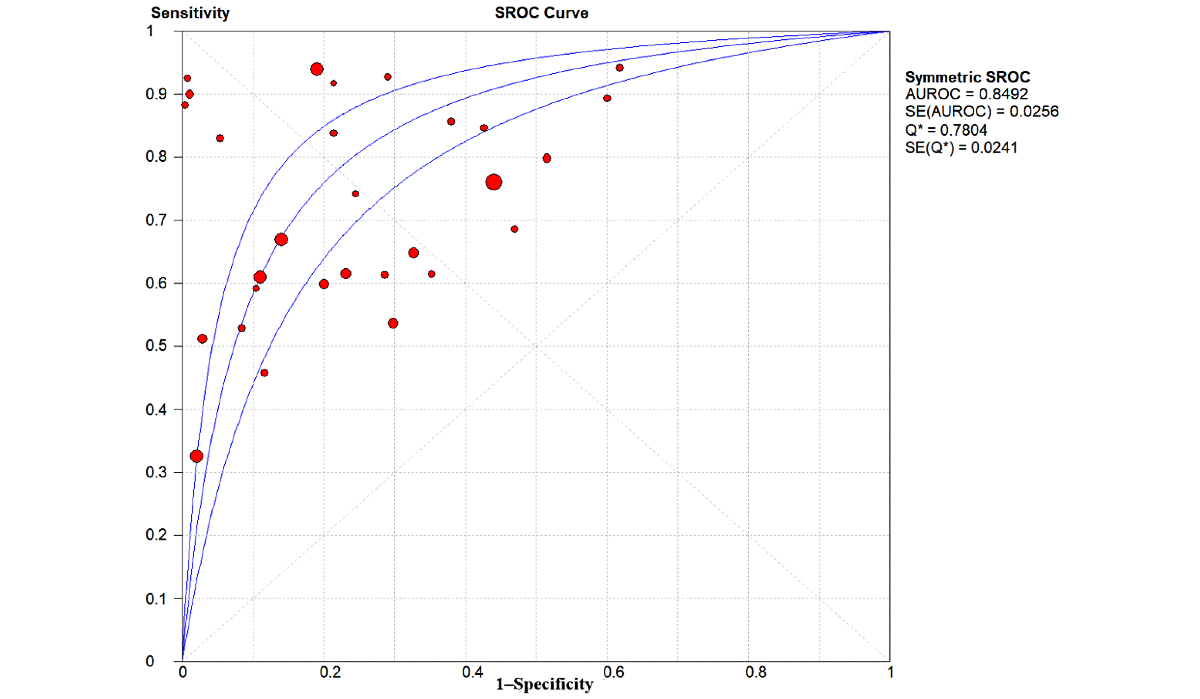
The overall pooled area under the receiver operating characteristic curve (AUROC) of machine learning models for gestational diabetes mellitus prediction. Q*: the sensitivity at the intersection of the SROC curve and the straight line (sensitivity=specificity); SROC: summary receiver operating characteristic.

**Figure 3:**
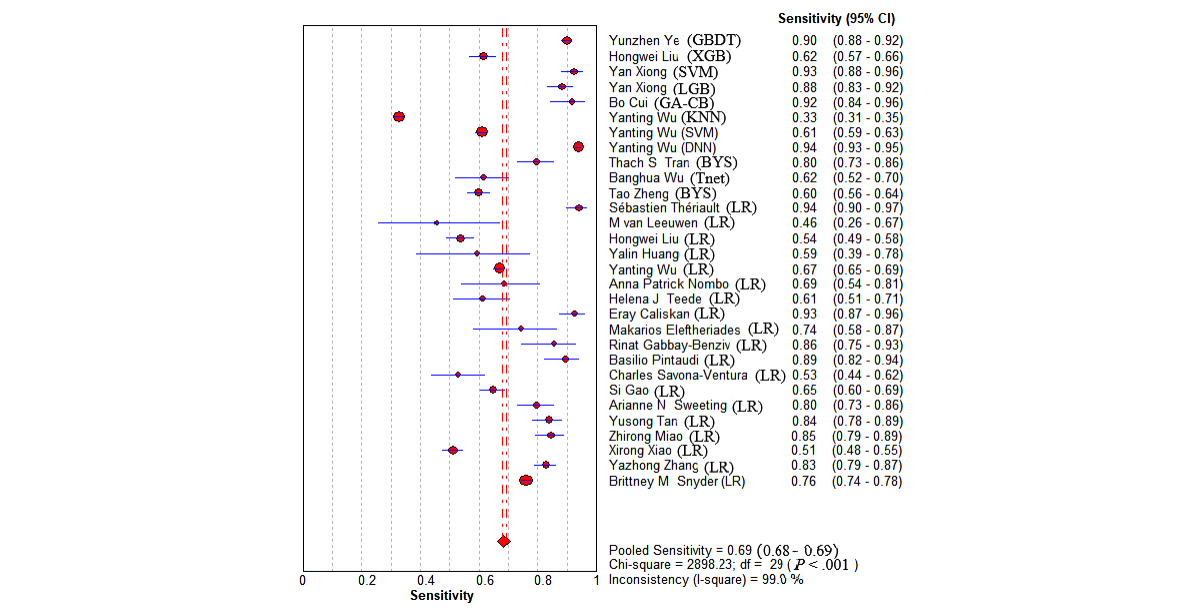
The overall pooled sensitivity of machine learning models for gestational diabetes mellitus prediction. First authors for each study are listed along the y-axis. The vertical red dotted lines are the 95% CIs of the pooled sensitivity. BYS: Bayesian; DNN: deep neural network; GA-CB: GA-CatBoost (genetic algorithm category boosting); GBDT: gradient-boosting decision tree; KNN: k-nearest neighbors; LGB: LightGBM (light gradient boosting machine); LR: logistic regression; SVM: support vector machine; Tnet: TreeNet; XGB: XGBoost (extreme gradient boosting).

**Figure 4:**
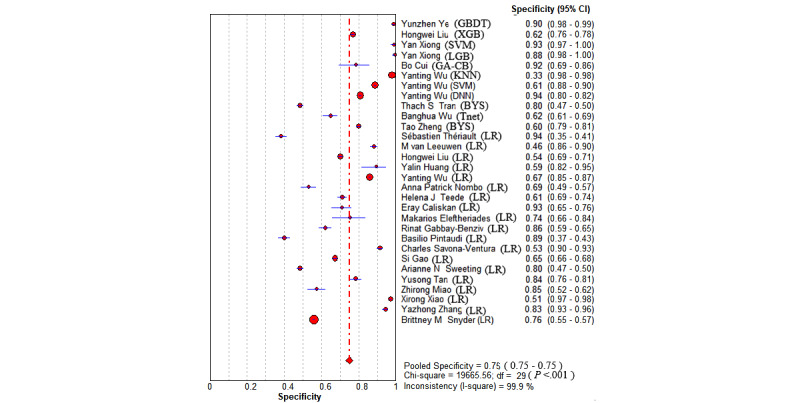
The overall pooled specificity of machine learning for gestational diabetes mellitus prediction. First authors for each study are listed along the y-axis. The vertical red dotted line is the 95% CI of the pooled specificity. BYS: Bayesian; DNN: deep neural network; GA-CB: GA-CatBoost (genetic algorithm category boosting); GBDT: gradient-boosting decision tree; KNN: k-nearest neighbors; LGB: LightGBM (light gradient boosting machine); LR: logistic regression; SVM: support vector machine; Tnet: TreeNet; XGB: XGBoost (extreme gradient boosting).

The overall pooled AUROC for ML models for predicting GDM was 0.8492 (Figure 2). Additional values were as follows: sensitivity 0.69 (95% CI 0.68-0.69; *P*<.001; I^2^=99.6%; Figure 3); specificity 0.75 (95% CI 0.75-0.75; *P*<.001; I^2^=100%; Figure 4); DOR 13.78 (95% CI 9.53-19.94; *P*<.001; I^2^=99.1%); PLR 4.02 (95% CI 3.13-5.17; *P*<.001; I^2^=99.6%); and NLR 0.31 (95% CI 0.26-0.38; *P*<.001; I^2^=98.7%).

### Sensitivity Analysis

After the exclusion of the 4 (16%) models with the lowest and highest sensitivity and specificity, the random effects meta-analysis produced estimated pooled sensitivity of 0.73 (95% CI 0.72-0.74; *P*<.001; I^2^=98.3%) and pooled specificity of 0.73 (95% CI 0.72-0.73; *P*<.001; I^2^=99.8%). Therefore, the pooled estimates were deemed insensitive to the exclusion of outliers (Figure S2 in Multimedia Appendix 1).

### Subgroup Analysis

The comparison of the GDM prediction performance results is shown in Table 2; forest plots are shown in Figures S3-S8 in Multimedia Appendix 1.

**Figure 5:**
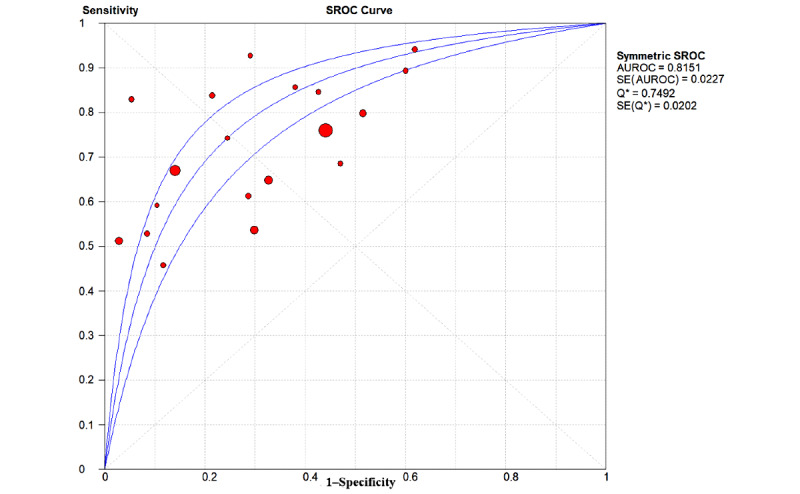
The overall pooled area under the receiver operating characteristic curve (AUROC) of logistic regression models for gestational diabetes mellitus prediction. Q*: the sensitivity at the intersection of the SROC curve and the straight line (sensitivity=specificity); SROC: summary receiver operating characteristic.

**Figure 6:**
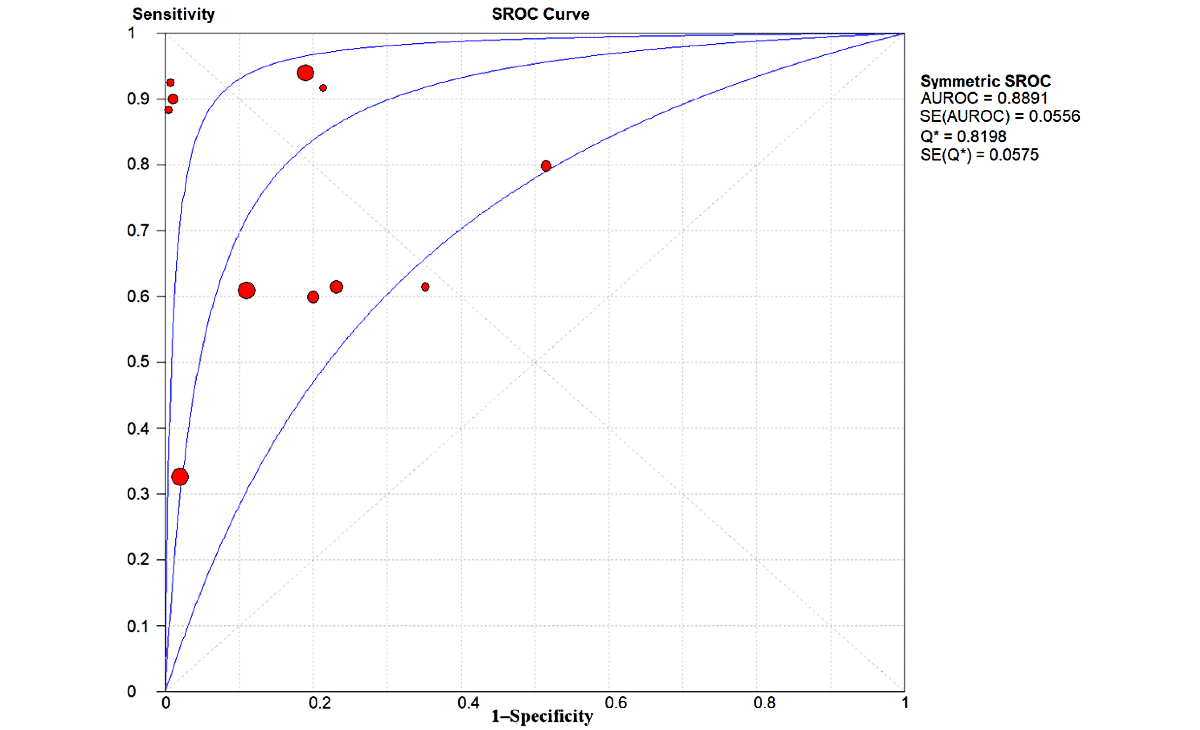
The overall pooled area under the receiver operating characteristic curve (AUROC) of non–logistic regression models for gestational diabetes mellitus prediction. Q*: the sensitivity at the intersection of the SROC curve and the straight line (sensitivity=specificity); SROC: summary receiver operating characteristic.

In this study, 19 prediction models were established using the LR models [17,18,20-23,26-33,35-39], and the overall pooled AUROC for the LR models for predicting GDM was 0.8151 (Figure 5). The overall pooled AUROC for non-LR models to predict GDM was 0.8891 (Figure 6), the highest value among these subgroups. Further analysis of these non-LR methods showed that two support vector machine (SVM) models [19,20] achieved AUROC values of 0.82 and 0.98, respectively (Figure S9 in Multimedia Appendix 1), while two Bayesian models [24,34] achieved AUROC values of 0.766 and 0.71, respectively (Figure S10 in Multimedia Appendix 1). Interestingly, Ye et al [20] developed eight common ML methods—GBDT, AdaBoost, LightGBM, LR, Vote, XGBoost, decision tree (DT), and RF—and two common regression models to predict the occurrence of GDM with a data set of 822,242 patients. GBDT, AdaBoost, and LightGBM (AUROC 0.70-0.75) were the top three models, while DT and RF were the worst models (AUROC 0.5-0.68) in that study. The capabilities of three ML methods were compared using data from 490 people [21]. The deep neural network model achieved the highest AUROC of 0.92, while the SVM and k-nearest neighbors (KNN) models achieved AUROC values of 0.82 and 0.68, respectively. XGBoost, LightGBM, GA-CatBoost, and TreeNet were used in 4 out of 25 (16%) studies and achieved AUROC values of 0.742, 0.942, 0.872, and 0.676, respectively [17,19,21,25] (Table 2).

**Table 2:** The comparison of performance of machine learning models in gestational diabetes mellitus (GDM) prediction applied to different subgroups.

Subgroup	Models (N=30), n (%)	AUROC^a^	Sensitivity (95% CI)	Specificity (95% CI)	PLR^b^ (95% CI)	NLR^c^ (95% CI)	DOR^d^ (95% CI)
Overall	30 (100)	0.8492	0.69 (0.68-0.69)	0.75 (0.75-0.75)	4.02 (3.13-5.17)	0.31 (0.26-0.38)	13.78 (9.53-19.94)
0-13 weeks before diagnosis	16 (53)	0.8667	0.74 (0.73-0.75)	0.64 (0.64-0.64)	3.89 (2.92-5.19)	0.28 (0.22-0.36)	16.55 (9.52-28.77)
14-28 weeks before diagnosis	14 (47)	0.8365	0.64 (0.63-0.65)	0.85 (0.84-0.85)	3.90 (2.76-5.53)	0.35 (0.25-0.48)	11.67 (7.59-18.02)
With GDM history	11 (37)	0.8759	0.67 (0.66-0.68)	0.85 (0.85-0.86)	5.29 (3.39-8.25)	0.28 (0.18-0.44)	19.82 (11.49-34.13)
Without GDM history	19 (63)	0.8330	0.70 (0.66-0.68)	0.65 (0.64-0.65)	3.12 (2.52-3.86)	0.35 (0.30-0.41)	8.27 (5.14-13.29)
Logistic regression	19 (63)	0.8151	0.71 (0.70-0.72)	0.67 (0.67-0.67)	3.04 (2.37-3.89)	0.37 (0.32-0.43)	8.73 (5.99-12.73)
Non–logistic regression	11 (37)	0.8891	0.66 (0.65-0.67)	0.85 (0.85-0.86)	6.80 (4.45-10.37)	0.24 (0.15-0.38)	31.85 (15.93-63.69)

^a^AUROC: area under receiver operating characteristic curve.

^b^PLR: positive likelihood ratio.

^c^NLR: negative likelihood ratio.

^d^DOR: diagnostic odds ratio.

### Meta-regression

The meta-regression analysis was conducted due to the high level of interstudy heterogeneity [42]. Sample size, country where the data were collected, publication year, ML methods used, and model quality did not affect diagnostic accuracy (*P*=.13). The antilogarithm transformations of the resulting estimated parameters could be interpreted as a relative DOR of the corresponding covariate, indicating the change in diagnostic performance of the test under study per unit increase in the covariate (Table S8 in Multimedia Appendix 1).

## Discussion

### Principal Findings

This study was a pilot meta-analysis evaluating the performance of ML models for predicting GDM. Its overall pooled estimation of 25 studies showed that ML models achieved high accuracy in early recognition of GDM patients. ML models could forecast based on data from 8 to 24 weeks’ gestation. There was even a model that used prepregnancy features to predict the outcome up to 28 weeks in advance, suggesting the significance of ML models for GDM prediction. Compared to the census or existing screening methods, ML methods have certain advantages. Universal screening leads to 100% detection for physicians who usually make decisions based on an OGTT test, which may place an unnecessary burden on individual women and health care resources. Current selective screening strategies are based on a list of risk factors and have fixed sensitivity (±65%) and specificity (±80%). Although the ML methods do not provide greater benefit than current available screening strategies, an advantage is that a preferred trade-off between sensitivity and specificity can be selected [43]. The choice of statistical method is more to compute a quantitative measure of existing data than to predict unknown data in a general and feasible way [44].

According to the subgroup analysis, models created using non-LR methods achieved the highest AUROC, suggesting that researchers should test more candidate models. One study aimed to review and compare the predictive performances of LR and other ML algorithms for developing or validating a multivariable prognostic prediction model for pregnancy care; that study also recommended a reanalysis of existing LR models for several pregnancy outcomes by comparing them with those algorithms that apply standard guidelines [45]. Among those non-LR models, ensemble methods, like LightGBM and GA-CatBoost, that are composed of multiple weaker models and are independently trained had a satisfactory result. Variables in the GBDT model underscored the advantage of identifying nonlinear relationships. The SVM model also achieved superior outcomes; that method builds a model that assigns new examples to one category or the other, making it a nonprobabilistic binary linear classifier. Methods like KNN, DT, and RF did not perform as well as the LightGBM and GA-CatBoost methods, which may be due to the fact that DT classifications are based on a single condition at the bottom, so small changes can lead to mistakes. For RF, the high dimension of medical data complicates the classification and prediction. Similarly, KNN cannot be used in high-dimensional feature spaces. Some researchers [23] found that the difference between two methods had no statistical significance, since LR models are suitable for simple data with linear relationships between variables and outcomes. Our study also found that LR models were conducive to achieving more stable performance according to the summary receiver operating characteristic curve. The subgroup of 0 to 13 weeks before diagnosis achieved the highest pooled sensitivity, while the subgroup of 14 to 28 weeks before diagnosis achieved the highest specificity, meaning that ML may assist clinicians identify more patients in early screening and avoid excessive misdiagnosis in the second trimester.

The feature selection was also crucial for model performance and interpretation. Among the 25 studies, maternal age was used as a feature in 19 studies, as was previously reported and validated in our study. One of the included studies reported that the incidence of GDM increases after 25 years of age, the main reason being that the function of islet β-cells decreases with age, so the insulin antagonism of older adult pregnant women is aggravated [46]. Eight models considered GDM history to be a vital factor for predicting GDM. A DOR value of 21.09 appeared when a GDM history was included as a risk factor for predicting future GDM. Previous research discovered that women with GDM were more likely to have a family history of type 2 diabetes mellitus and a history of GDM, partially due to overlapping genetic bases between the diseases [18]. The nonsignificant association of GDM with a GDM history in other studies was a result of the overwhelming proportion of nulliparous women in their studies who had no risk of developing GDM. The association between GDM and blood lipid indexes, including triglyceride (TG), high-density lipoprotein, and low-density lipoprotein (LDL), has been studied, and TG level had the closest relationship with GDM [47]. Our research also found that although the levels of TG, total cholesterol, and LDL in the GDM group were higher than those in normal pregnant women in most included studies, only TG level was a high-risk factor of GDM after the feature selection. A novel model that included ultrasound data of maternal fat distribution and serum inflammatory factors observed that pregnant women with GDM had greater visceral fat thickness and subcutaneous fat thickness; the model also demonstrated that increased subcutaneous and visceral fat may lead to increased insulin resistance in muscle and adipose tissue [36]. Sweeting et al [27] observed higher leptin and lipocalin-2 levels and lower adiponectin levels in women who developed GDM and proposed adipokines as GDM features.

### Strengths and Limitations

The main strength of the study is that its methodology was logical and described in sufficient detail to be reproducible. Almost all published prognostic models for GDM were included in this meta-analysis, which enabled their comparison. The data collection table was based on the characteristics of the GDM prediction models. Additionally, the novel PROBAST was used to assess the risk of bias and applicability of prognostic prediction model studies. The Quality Assessment of Diagnostic Accuracy Studies (QUADAS) tool is a widely used tool for estimating the bias and applicability of primary diagnostic accuracy studies, but is not perfectly suited for predictive models [48]. An increasing number of researchers prefer the PROBAST to the QUADAS tool for assessing the bias of AI-based models in systematic reviews as well as meta-analyses; this is the case because more details of the model, such as data source, processing, number of events per variable, feature selection, model development, and model validation, were checked intensively [49-52]. We found that 14 development studies had a risk of bias in methodological quality or applicability, which may lead to overfitted prediction models. It is noteworthy that the quality of recent models is higher than that of those published earlier according to the PROBAST. Some bias could be prevented if the studies reported their research according to the TRIPOD initiative [12].

Despite our study’s confirmation that ML models have promising prediction ability for GDM, there are some limitations to our research. The main limitations arose from the interstudy heterogeneity. First, the sample sizes and distributions differed among studies, affecting each model’s performance and applicability. There were also a heterogenous variety of feature selection methods. Some researchers preferred the features that have a statistically significant association with GDM, while others included the factors based on existing knowledge from previously established models in combination with predictor reliability, consistency, applicability, availability, and cost. Second, the performance of a low-quality model might be overestimated when the analysis of the internal bias of the model is ignored. As some studies have bias to various degrees, the results of the studies in this analysis must be applied with caution. It should be noticed that the PROBAST is more likely to identify bias in prediction models than other tools designed for conventional diagnostic methods. The other limitation is that few models underwent external validation to test their extensibility. However, a previous study [8] performed an external validation of 12 published GDM prediction models and suggested that most of the published models showed acceptable discrimination and calibration, but the author pointed out possible heterogeneity in these models due to variations in GDM incidence in different populations.

### Clinical Implications

Although several GDM scoring systems have been developed, none are widely recommended by current guidelines. Based on the discussion above, several items must be considered in order to maximize the advantages of ML models for predicting GDM in clinical practice for model researchers or for decision makers. For the former, we recommend that the decision concerning which feature selection methods and ML algorithms to use should be based on clinical need rather than accuracy. A model with excess features that are difficult to obtain in routine medicine is unlikely to be applied broadly. Researchers should also provide the process of data preprocessing and outcomes of validation, discrimination, calibration, and classification to elaborate the performance of models from multiple perspectives. For decision makers, we recommend that data sources, such as a population-based cohort designed for GDM research with a unified international diagnostic criterion, promote the ML methods in this target. Studies revealed that although electronic health records provide various data, including time series and images for novel ML methods, they have inherent biases that are influenced by the interaction of the patient with the health care system. In contrast, community-based predictions may robustly capture more asymptomatic high-risk cases [53]. The incidence of GDM based on the International Association of the Diabetes and Pregnancy Study Groups (IADPSG) (22.94%) and the National Institute for Health and Care Excellence (21.72%) is over 3-fold higher than that based on the criteria from the 7th edition of the Chinese obstetrics and gynecology textbook (6.08%) published by the People’s Medical Publishing House [54]. Some experts in China have advocated implementation of the IADPSG criteria because they believe that it will guide researchers to better understand the prevalence of GDM in different regions and ensure that the country’s standards will be aligned with international ones. Nevertheless, researchers doubt that the IADPSG findings will apply to all populations, since those criteria were applied to mainly Caucasian women. All in all, it would indeed be helpful to unify the GDM diagnostic criteria as soon as possible. This meta-analysis reported the advantages of ML models and the factors requiring attention. A similar meta-analysis of ML models and deep learning algorithms used to detect patients at risk of developing diabetes reported that AI-based automated tools provide substantial benefits for reducing screening costs and can replace earlier treatments [55].

## Conclusions

In conclusion, ML methods demonstrate high performance and will be a more selective and cost-effective screening method for GDM. The importance of quality assessment and unified diagnostic criteria should be further emphasized.

## Appendix

Multimedia Appendix 1 Supplementary materials.

Multimedia Appendix 2 Checklists from the Prediction Model Risk of Bias Assessment Tool (PROBAST).

## Abbreviations

AdaBoost: adaptive boosting
AI: artificial intelligence
AUROC: area under the receiver operating characteristic curve
C-index: concordance index
DOR: diagnostic odds ratio
DT: decision tree
FDA: Food and Drug Administration
GA-CatBoost: genetic algorithm category boosting
GBDT: gradient-boosting decision tree
GDM: gestational diabetes mellitus
IADPSG: International Association of the Diabetes and Pregnancy Study Groups
KNN: k-nearest neighbors
LDL: low-density lipoprotein
LightGBM: light gradient boosting machine
LR: logistic regression
ML: machine learning
NLR: negative likelihood ratio
OGTT: oral glucose tolerance test
PICO: population, intervention, control, and outcomes
PLR: positive likelihood ratio
PRISMA: Preferred Reporting Items for Systematic Reviews and Meta-Analyses
PROBAST: Prediction Model Risk of Bias Assessment Tool
QUADAS: Quality Assessment of Diagnostic Accuracy Studies
RF: random forest
SVM: support vector machine
TG: triglyceride
TRIPOD: Transparent Reporting of a Multivariable Prediction Model for Individual Prognosis or Diagnosis
XGBoost: extreme gradient boosting
