# Supplementary material for: Machine Learning Prediction Models for Gestational Diabetes Mellitus: Meta-analysis
Source: J Med Internet Res. 2022 Mar 16;24(3):e26634. doi: 10.2196/26634 (PMC8968560; doi:10.2196/26634)
Supplement: Multimedia Appendix 1 [file jmir_v24i3e26634_app1.docx]

Multimedia Appendix 1

(Machine Learning Prediction Models for Gestational Diabetes Mellitus: Meta-analysis)

##### Table S1: Summary of international diagnostic criteria for GDM

| Criteria | Year | Fasting | | 1-hour | | 2-hour | | 3-hour | |
| --- | --- | --- | --- | --- | --- | --- | --- | --- | --- |
|  |  | mg/dL | mmol/L | mg/dL | mmol/L | mg/dL | mmol/L | mg/dL | mmol/L |
| ADA^1,2^ | 2003 | 95 | 5.3 | 180 | 10.0 | 155 | 8.6 | 140 | 7.8 |
| ADIPS^3^ | 2014 | 92 | 5.1 | 180 | 10.0 | 153 | 8.5 | - | - |
| Diabetes Canada Clinical Practice Guidelines^4^ | 2018 | 95 | 5.3 | - | 10.6 | - | 9.0 | - | - |
| DIPSI^5^ | 2014 | - | - | - | - | 140 | 7.8 | - | - |
| IADPSG^4^ | 2010 | 92 | 5.1 | 180 | 10.0 | 153 | 8.5 | - | - |
| WHO^6^ | 2013 | 92 | 5.1 | 180 | 10.0 | 153 | 8.5 | - | - |
| NDDG^7^ | 1979 | 95 | 5.8 | 180 | 10.5 | 155 | 9.1 | 140 | 8.0 |

Abbreviations: ADA: American Diabetes Association; ADIPS: Australasian Diabetes in Pregnancy Society; DIPSI: Diabetes Canada Clinical Practice Guidelines Diabetes in Pregnancy Society Group India; IADPSG: International Association of the Diabetes and Pregnancy Study Groups; WHO: World Health Organization; NDDG: National Diabetes Data Group.

1. American Diabetes Association. Gestational diabetes mellitus. Diabetes Care. 2003;26(S1): S103– 5.

2. American Diabetes Association. Classification and diagnosis of diabetes: Standards of medical care in diabetes – 2018. Diabetes Care. 2018;41(Suppl 1):S13–27

3. A N, HD M, R M et al. ADIPS Consensus Guidelines for the Testing and Diagnosis of Hyperglycaemia in Pregnancy in Australia and New Zealand. 2014;

4. Diabetes Canada Clinical Practice Guidelines Expert Committee, DS F, H B et al. Diabetes and pregnancy. Can J Diabetes. 2018;(42):S255–82

5. V S, S B, V B et al. Consensus evidence-based guidelines for management of gestational diabetes mellitus in India. Journal of the Association of Physicians of India. 2014;62(7 Suppl):55-62

6. World Health Organization. Diagnostic criteria and classification of hyperglycaemia first detected in pregnancy: a World Health Organization Guideline. Diabetes Res Clin Pract. 2014;103(3):341-63

7. National Diabetes Data Group, GROUP NDD. Classification and diagnosis of diabetes mellitus and other categories of glucose intolerance. Diabetes. 1979;28(12):1039-1057

##### Table S2: PRISMA checklists^8^

| **Section/topic** | **#** | **Checklist** | **Reported on page #** |
| --- | --- | --- | --- |
| **TITLE** | | | |
| Title | 1 | Identify the report as a systematic review, meta-analysis, or both. | 1 |
| **ABSTRACT** | | |  |
| Structured summary | 2 | Provide a structured summary including, as applicable: background; objectives; data sources; study eligibility criteria, participants, and interventions; study appraisal and synthesis methods; results; limitations; conclusions and implications of key findings; systematic review registration number. | 2 |
| **INTRODUCTION** | | | |
| Rationale | 3 | Describe the rationale for the review in the context of what is already known. | 3 |
| Objectives | 4 | Provide an explicit statement of questions being addressed with reference to participants, interventions, comparisons, outcomes, and study design (PICOS). | Table S3 |
| **METHODS** | | | |
| Protocol and registration | 5 | Indicate if a review protocol exists, if and where it can be accessed (e.g., Web address), and, if available, provide registration information including registration number. | / |
| Eligibility criteria | 6 | Specify study characteristics (e.g., PICOS, length of follow-up) and report characteristics (e.g., years considered, language, publication status) used as criteria for eligibility, giving rationale. | 5 |
| Information sources | 7 | Describe all information sources (e.g., databases with dates of coverage, contact with study authors to identify additional studies) in the search and date last searched. | 4 |
| Search | 8 | Present full electronic search strategy for at least one database, including any limits used, such that it could be repeated. | 4,5 |
| Study selection | 9 | State the process for selecting studies (i.e., screening, eligibility, included in systematic review, and, if applicable, included in the meta-analysis). | 6,7 |
| Data collection process | 10 | Describe method of data extraction from reports (e.g., piloted forms, independently, in duplicate) and any processes for obtaining and confirming data from investigators. | 5 |
| Data items | 11 | List and define all variables for which data were sought (e.g., PICOS, funding sources) and any assumptions and simplifications made. | 5, Table S3 |
| Risk of bias in individual studies | 12 | Describe methods used for assessing risk of bias of individual studies (including specification of whether this was done at the study or outcome level), and how this information is to be used in any data synthesis. | 5,6 |
| Summary measures | 13 | State the principal summary measures (e.g., risk ratio, difference in means). | 6 |
| Synthesis of results | 14 | Describe the methods of handling data and combining results of studies, if done, including measures of consistency (e.g., I^2^) for each meta-analysis. | 6 |
| Risk of bias across studies | 15 | Specify any assessment of risk of bias that may affect the cumulative evidence (e.g., publication bias, selective reporting within studies). | 6 |
| Additional analyses | 16 | Describe methods of additional analyses (e.g., sensitivity or subgroup analyses, meta-regression), if done, indicating which were pre-specified. | 6 |
| **RESULTS** | | | |
| Study selection | 17 | Give numbers of studies screened, assessed for eligibility, and included in the review, with reasons for exclusions at each stage, ideally with a flow diagram. | 6, 7, Figure 1 |
| Study characteristics | 18 | For each study, present characteristics for which data were extracted (e.g., study size, PICOS, follow-up period) and provide the citations. | 7-9, Table S4-S6 |
| Risk of bias within studies | 19 | Present data on risk of bias of each study and, if available, any outcome level assessment (see item 12). | 11,12, Table 4 |
| Results of individual studies | 20 | For all outcomes considered (benefits or harms), present, for each study: (a) simple summary data for each intervention group (b) effect estimates and confidence intervals, ideally with a forest plot. | Table S4-S6 |
| Synthesis of results | 21 | Present results of each meta-analysis done, including confidence intervals and measures of consistency. | 12, Figure S1-S6 |
| Risk of bias across studies | 22 | Present results of any assessment of risk of bias across studies (see Item 15) | 12 |
| Additional analysis | 23 | Give results of additional analyses, if done (e.g., sensitivity or subgroup analyses, meta-regression [see Item 16]). | 13-15, Table5, Table6, Table 7, Figure S7-S56 |
| **DISCUSSION** | | | |
| Summary of evidence | 24 | Summarize the main findings including the strength of evidence for each main outcome; consider their relevance to key groups (e.g., healthcare providers, users, and policy makers). | 13-16 |
| Limitations | 25 | Discuss limitations at study and outcome level (e.g., risk of bias), and at review-level (e.g., incomplete retrieval of identified research, reporting bias). | 16 |
| Conclusions | 26 | Provide a general interpretation of the results in the context of other evidence, and implications for future research. | 16-17 |
| **FUNDING** | | | |
| Funding | 27 | Describe sources of funding for the systematic review and other support (e.g., supply of data); role of funders for the systematic review. | 17 |

8. Wolff RF, Moons KGM, Riley RD et al. PRISMA. Ann Intern Med. 2019;170(1):51-58

##### Textbox S1: Search terms

| The following terms were used to search the articles in PubMed. It should be noted that we initially screened all diabetes-related complications and decided to focus on gestational diabetes finally.  ((diabet* OR “type 2 diabetes” OR T2D* OR “type 1 diabetes mellitus” OR “undiagnosed diabetes” OR “prevalent diabetes” OR “hyperglycemia”) [All Fields] AND (predict* OR estimate* OR evaluat* OR identif* OR diagnos* OR screen*) [All Fields] AND (risk* OR “risk factor” OR “future risk” OR “high risk model” OR “risk model” OR “risk adjusted models” OR inciden*)[All Fields] AND (“logistic regression” OR “proportional hazards model” OR “deep learning OR “ML” OR “support vector machine” OR “decision tree” OR “random forest” OR “k-nearest neighbor” OR “recurrent neural network” OR “convolutional neural network” OR “boost*”) [All Fields]). |
| --- |

##### Table S3: Selection criteria of predictive modelling studies in PICOTS format

|  | **Participants (P)** | **Intervention**  **(I)** | **Control (C)** | **Outcomes**  **(O)** | **Timeframe**  **(T)** | **Setting**  **(S)** | **Other limits** |
| --- | --- | --- | --- | --- | --- | --- | --- |
| **Inclusion criteria** | Pregnant women>18；  Women plan to get pregnant >18 | ML predictive modelling: supervised, unsupervised, semi-supervised ML or combinations | Golden standards | **Primary:** metrics of discrimination ability, calibration, and classification accuracy in GDM prediction  **Secondary:** important variables, intended use of models | 2004-2020 | **Clinical care settings** e.g. hospitals, institutions, case-control study, population-based cohort. | Language = English, Chinese |
| **Exclusion criteria** | Patients with other clinical phenotypes of diabetes; Diabetic complication;  Woman of high-risk known | Predictive modelling without an explicit ML approach; Diagnostic models |  |  |  |  | Other language |

##### Table S4: Baseline characteristics of the 25 studies selected for the meta-analysis (data sources and participants)

| Study ID | Country in which data was gathered | Setting | Data source | Study design | Prediction temporality | Primary outcome definition | Included T2DM cases |
| --- | --- | --- | --- | --- | --- | --- | --- |
| Tan2020 | China | Hospital-based | EHRs, questionnaires | Retrospective cohort | Prognostic | NDDG | GDM/non-GDM |
| Teede2011 | Australia | Hospital-based | EHRs | Retrospective cohort | Prognostic | NR | GDM/non-GDM |
| Tran2013 | Vietnam | Hospital-based | EHRs | A prospective cross-sectional study | Prognostic | IADPSG | Unknown GDM |
| Van Leeuwen2009 | Netherlands | Hospital-based | EHRs | A prospective cohort study | Prognostic | Carpenter-Coustan thresholds | Unknown GDM |
| Wu2017 | China | Hospital-based | EHRs | Retrospective | Prognostic | IADPSG | GDM/non-GDM |
| Wu2020 | China | Hospital-based | EHRs | Retrospective | Prognostic | NR | GDM/non-GDM |
| Xiao2018 | China | Hospital-based | EHRs | Retrospective | Prognostic | IADPSG | GDM/non-GDM |
| Xiong2020 | China | Hospital-based | EHRs | Case-control study | Prognostic | IADPSG | GDM/non-GDM |
| Ye2020 | China | Hospital-based | EHRs | Retrospective cohort | Prognostic | WHO | GDM/non-GDM |
| Zhang2020 | China | Hospital-based | EHRs, questionnaires | Retrospective cohort | Prognostic | IADPSG | GDM/non-GDM |
| Zheng2019 | China | Hospital-based | EHRs | Retrospective and prospective birth cohort | Prognostic | ADA | GDM/non-GDM |
| Eleftheriades2014 | Greece | Hospital-based | EHRs | Case control prospective observational study | Prognostic | ICD-9-CM diagnostic codes | GDM/non-GDM |
| Gabbay-Benziv2015 | America | Multicentre | EHRs, questionnaire | Prospective cohort study for placental dysfunction | Prognostic | ADIPS | GDM/non-GDM |
| Gao2020 | China | Population-based | Questionnaires, | Prospective cohort | Prognostic | FPG: 5. 1 mmol /L， OGTT 1 h:10. 0 mmol /L， OGTT 2h: 8. 5 mmol /L) | Unknown GDM |
| Liu2020 | China | population-based | Questionnaires | Prospective cohort | Prognostic | FPG: 5.5 mmol⁄L or a 2-h level of 8.0 mmol⁄L | Unknown GDM |
| Miao2020 | China | Hospital-based | EHRs, telephone follow-up | Prospective case control | Prognostic | CDA | Unknown GDM |
| Huang2017 | China | Hospital-based | EHRs | Birth cohort | Prognostic | ADA | Unknown GDM |
| Nombo  2018 | Tanzania | Multicentre | EHRs | Cross-sectional | Prognostic | WHO | Unknown GDM |
| Pintaudi  2014 | Italy | Hospital-based | EHRs | Retrospective study | Prognostic | NR | Unknown GDM |
| Savona-Ventura  2013 | Malta; Greece; Serbia; Italy France; Portugal; Morocco; Tunisia; Algeria, Syria; Lebanon | Multicentre study | EHRs | Prospective, non-interventional study | Prognostic | IADPSG | GDM/non-GDM |
| Thériault  2015 | Canada | Hospital-based | Questionnaire | Prospective case-control cohort | Prognostic | FPG: ≥5.1mmol/L ;1 h OGTT≥10 mmol/L;2 h OGTT≥8.5 mmol/L | GDM/non-GDM |
| Snyder  2020 | America | Population-based | Population-based cohort | Population-based cohort | Prognostic | WHO | Unknown GDM |
| Sweeting  2018 | Australia | Hospital-based | EHRs | Case-control study | Prognostic | IADPSG | GDM/non-GDM |
| Caliskan  2004 | Turkish | Population-based | EHRS and telephone follow-up | Population-based，retrospective case–control study | Prognostic | ADA | GDM/non-GDM |
| Cui2019 | China | Hospital-based | EHRS | Hospital-based cohort | Prognostic | IADPSG | GDM/non-GDM |

Abbreviations: EHRs: electronic health records; ADA: American Diabetes Association; ADIPS: Australasian Diabetes in Pregnancy Society; DIPSI: Diabetes Canada Clinical Practice Guidelines Diabetes in Pregnancy Society Group India; IADPSG: International Association of the Diabetes and Pregnancy Study Groups; WHO: World Health Organization; NDDG: National Diabetes Data Group; NR: not reported

##### Table S5: Baseline characteristics of the 25 studies selected for the meta-analysis (features and pre-processing)

| Study ID | Number of variables included | Missing data described | Missingness handling described | Feature selection algorithms | Pre-processing described | Feature selection described | ML algorithms |
| --- | --- | --- | --- | --- | --- | --- | --- |
| Caliskan  2004 | 5 | NO | NO | Logistic regression | YES | YES | Logistic regression |
| Cui2019 | 17 | YES | YES | Information value | YES | YES | Cat-boost |
| Eleftheriades2014 | 3 | NO | NO | Logistic regression | YES | YES | Logistic regression analysis with backward  elimination |
| Gabbay-Benziv2015 | 5 | NO | NO | Multivariable logistic regression with backward stepwise elimination | YES | YES | Multivariate logistic regression |
| Gao2020 | 10 | NO | NO | Univariate significance level, clinical importance, multivariate logistic regression | YES | YES | Multivariate logistic regression |
| Huang2017 | 7 | NO | YES | Multivariate logistic regression | YES | YES | Multivariate logistic regression |
| Liu2020 | 5 | NO | NO | Univariate backward selection; XGBoost | YES | YES | Multivariate logistic regression; XGBoost |
| Miao2020 | 7 | YES | YES | Univariate logistic regression | YES | YES | Multivariate logistic regression |
| Nombo2018 | 3 | NO | NO | Multivariate model | YES | YES | Multivariate model |
| Pintaudi2014 | 4 | NO | NO | Multivariate logistic regression analysis, RECPAM | YES | YES | Multivariate logistic regression analysis |
| Savona-Ventura2013 | 2 | NO | NO | Logistic multivariate analysis | YES | YES | Logistic multivariate analysis |
| Snyder2020 | 7 | YES | YES | NR | YES | YES | Logistic regression analysis |
| Sweeting2018 | 13 | NO | NO | Stepwise selection of variables | YES | YES | Multivariate logistic regression analysis |
| Tan2020 | 8 | NO | NO | Multivariate Logistic regression | NO | YES | Multivariate logistic regression |
| Teede2011 | 5 | NO | NO | Univariate analysis | NO | YES | Logistic regression |
| Thériault2015 | 3 | NO | YES | Bootstrap samples confirming the robustness of the selection procedure | YES | YES | Logistic regression analysis |
| Tran2013 | 2 | NO | NO | Bayesian model averaging | NO | YES | Bayesian model averaging |
| Van Leeuwen2009 | 4 | NO | YES | Univariable analysis, multiple logistic regression analysis | YES | YES | Multiple logistic regression analysis |
| Wu2017 | 37 | NO | NO | TreeNet | YES | YES | TreeNet, Cart |
| Wu2020 | 7/73 | NO | YES | RBF kernel Support 143 Vector Machine (SVM) classifier; K-Nearest Neighbour (KNN) classifier | YES | YES | Support vector machine (SVM), K-Nearest Neighbour (KNN) |
| Xiao2018 | 6 | YES | NO | Logistic regression | NO | NO | Logistic regression |
| Xiong2020 | 2 | NO | NO | Correlation | YES | YES | Support vector machine (SVM) and light gradient boosting machine (lightGBM) |
| Ye2020 | 8 | NO | YES | Pearson correlation  coefficient, Logistic regression | YES | YES | ML methods |
| Zhang2020 | 15 | NO | NO | Forward stepwise variable-selection approach | NR | YES | Logistic regression analysis |
| Zheng2019 | 4 | YES | YES | Univariate logistic regression | YES | YES | Multivariate Bayesian logistic regression |

Abbreviations: NR: not reported, ML: machine learning

##### Table S6: Baseline characteristics of the 25 studies selected for the meta-analysis (validation and application)

| Study ID | Internal validation | External validation | Model evaluation metrics | Class imbalance (GDM/Non-GDM) | Tools developed | funding | Ethics approval |
| --- | --- | --- | --- | --- | --- | --- | --- |
| Tan2020 | YES | NR | AUC, SPE | 143：286 | Model | YES | NR |
| Teede  2011 | NR | YES | SEN, SPE, PPV, NPV, AUC | 250:2630 | Scoring system | YES | NR |
| Tran2013 | YES | NR | AUC | 40：94 | Predictive nomogram | YES | YES |
| Van Leeuwen2009 | YES | NR | AUC, calibration | 63:861 | Clinical prediction rule, Nomogram | YES | YES |
| Wu2017 | YES | YES | TP, TN, FP, FN, ACC, AUC | 979:11980 | Rule | NR | NR |
| Wu2020 | YES | YES | AUC, SEN, SPE, | 114:464 | Model | YES | YES |
| Xiao2018 | YES | NR | AUC, SEN, NPV | 771:3153 | Model | NR | NR |
| Xiong  2020 | YES | NR | AUC | 222：420 | Model | YES | NR |
| Ye2020 | YES | NR | AUC | 3182: 19060 | Model | YES | YES |
| Zhang  2020 | NR | NR | AUC | 113:902 | Model | YES | YES |
| Zheng2019 | NR | NR | AUC, ACC, SEN, SPE, PPV, NPV | 119:1249 | Model | YES | YES |
| Eleftheriades2014 | NR | NR | AUC | 2520：30731 | Model | YES | YES |
| Gabbay-Benziv  2015 | NR | NR | AUC, SEN, SPE, PPV, NPV | 248:732 | Model | YES | YES |
| Gao2020 | YES | NR | AUC, SEN, SPE, PPV, NPV | 217：780 | Risk scores | YES | YES |
| Liu2020 | YES | NR | AUC, SEN, SPE, ACC, FP, FN | 250:2630 | Model | YES | YES |
| Miao  2020 | NR | NR | AUC, Yoden index | 222:420 | Model | NR | NR |
| Huang  2017 | YES | NR | AUC, SEN, SPE, PPV, NPV | 164:2608 | Model | NR | NR |
| Nombo  2018 | NR | NR | SEN, SPE, PPV, NPV, PLR, NLR | 2.4%：97.6% | Model | NR | YES |
| Pintaudi  2014 | NR | NR | OR, SEN, SPE, PPV, NPV | 798：5036 | Model | NO | YES |
| Savona-Ventura  2013 | NR | NR | AUC, SEN, SPE | 2696：14123 | Model | YES | YES |
| Thériault2015 | YES | NR | AUC | 771：3153 | Model | YES | NR |
| Snyder 2020 | YES | YES | AUC，SEN, SPE | 215:275 | Model | YES | NR |
| Sweeting2018 | NR | NR | AUC | 3182:19060 | Model | NR | YES |
| Caliskan2004 | NR | NR | SEN, SPE, PPV, NPV | 406：730 | Model | NR | NR |
| Cui2019 | YES | NR | ACC, PRE, RECALL, F1, AUC | 612:4159 | Model | NR | NR |

AUC: area under receiver operating characteristic curve; SPE: specificity; SEN: sensitivity, PPV: positive predictive value; NPV: negative predictive value, OR: odds ratio; TP: true positive; TN: true negative; FP: false positive; FN: false negative; PRE: precision; NR: not reported

##### Table S7: Risk of bias and applicability assessment by PROBAST criteria.

| Study | ROB | | | | Overall bias rating | Overall applicability rating |
| --- | --- | --- | --- | --- | --- | --- |
|  | Participants | Predictors | Outcome | Analysis |  |  |
| gao2020 | low | low | low | moderate | moderate | moderate |
| Liu2020 | low | low | low | low | low | low |
| miao2020 | low | low | low | low | low | low |
| tan2020 | low | low | low | low | low | low |
| Wu2020 | low | low | low | low | low | low |
| Yan2020 | low | low | low | low | low | low |
| Ye2020 | low | low | low | low | low | low |
| Zhang2020 | low | low | low | low | low | moderate |
| Snyder2020 | low | low | low | low | low | low |
| Cui2019 | low | low | low | low | low | moderate |
| Zheng2019 | low | low | low | low | low | low |
| Nombo2018 | low | low | low | low | low | low |
| Sweeting2018 | low | low | low | moderate | moderate | moderate |
| Xiao2018 | low | low | low | low | low | low |
| Huang2017 | low | low | low | moderate | moderate | low |
| Wu2017 | low | low | moderate | low | moderate | moderate |
| Gabbay-benziv2015 | moderate | low | moderate | high | high | low |
| Thériault2015 | low | low | low | low | low | moderate |
| Eleftheriades  2014 | moderate | low | low | moderate | moderate | low |
| Pintaudi2013 | low | moderate | low | moderate | moderate | low |
| Savonaventura2013 | moderate | low | moderate | moderate | moderate | moderate |
| Tran2013 | low | low | low | low | low | low |
| Teede2011 | low | low | moderate | low | moderate |  |
| Van leeuwen  2009 | moderate | low | moderate | moderate | moderate | low |
| Caliskan  2004 | low | low | moderate | moderate | moderate | low |

* When a single study included multiple models, risk of bias and applicability concerns were assessed for each model. Overall ratings per study have been presented since any discrepancies or counter-intuitive case scenarios with contradictory ratings between models within any single study were not observed.

##### Figure S1：The trend of published articles (articles included)


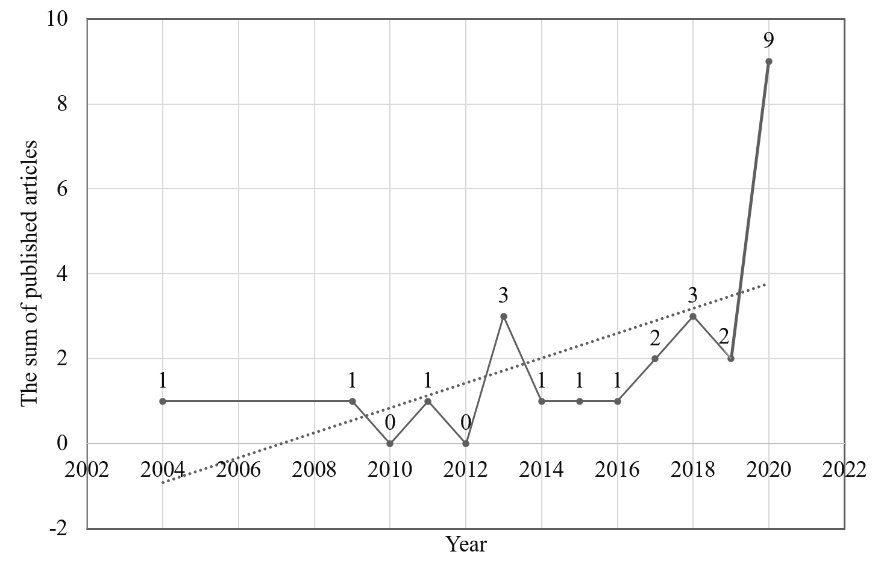


##### Figure S2: Forest plots of sensitivity analysis


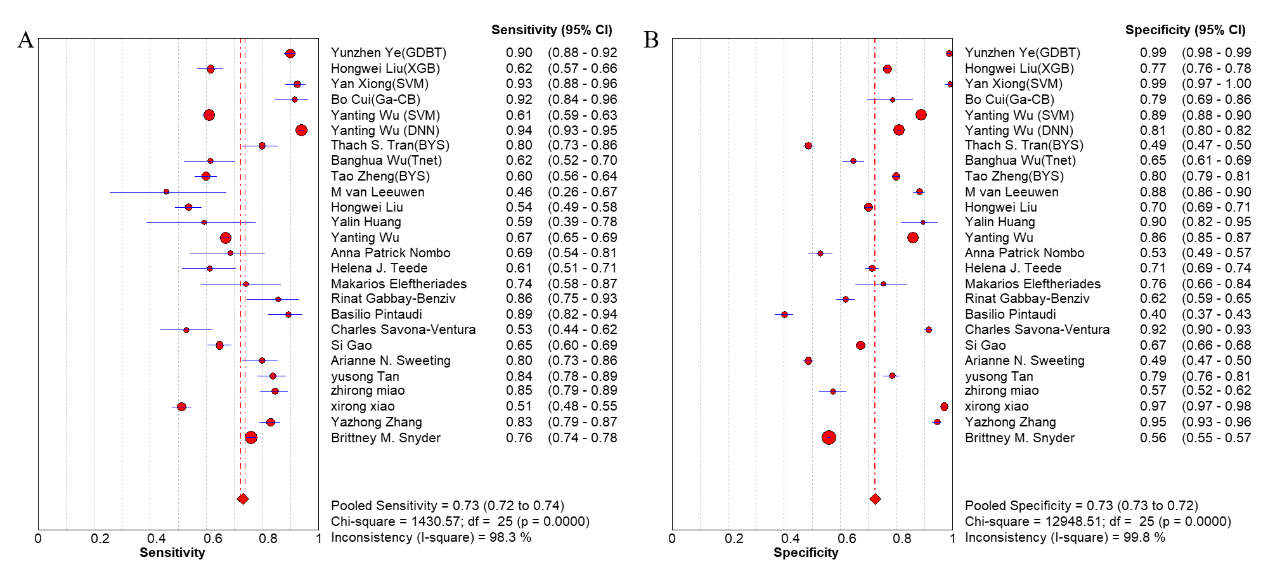


##### Figures S3-S10: Forest plots of machine learning model for predicting GDM (different subgroups).

**
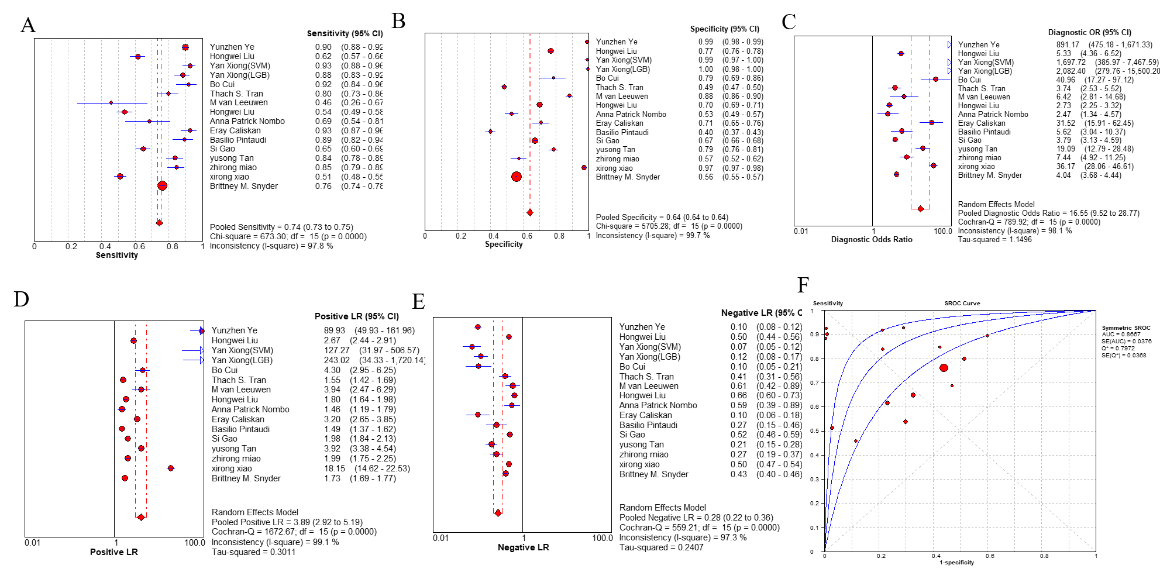
**

Fig.S.3. A-F. predict GDM 0-13 weeks before diagnosed

**
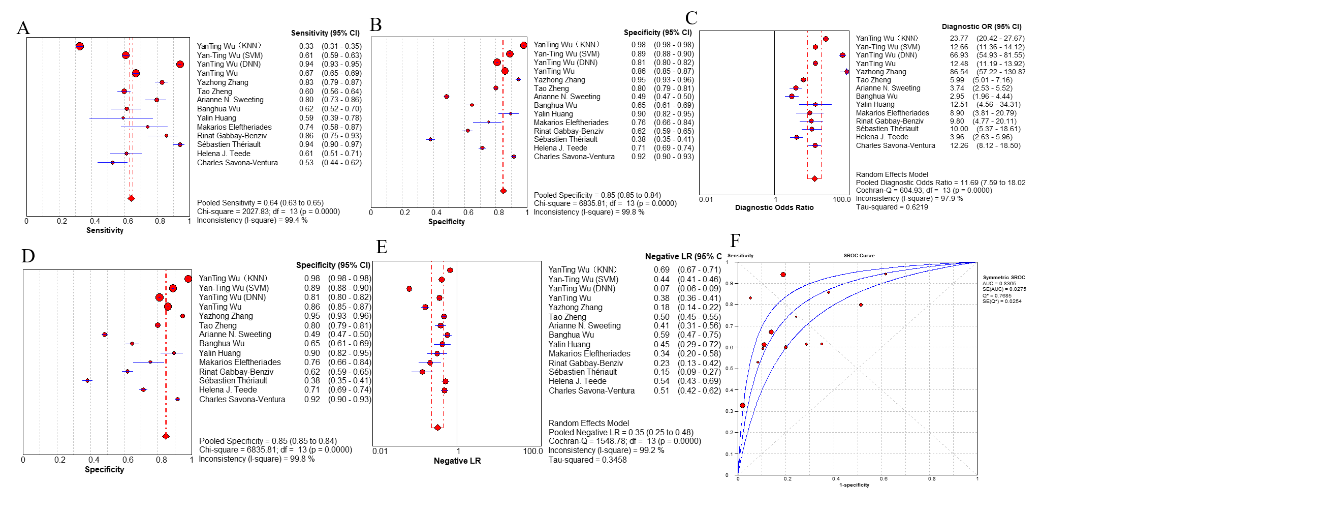
**

Fig.S.4. A-F. predict GDM 14-28 weeks before diagnosed

**
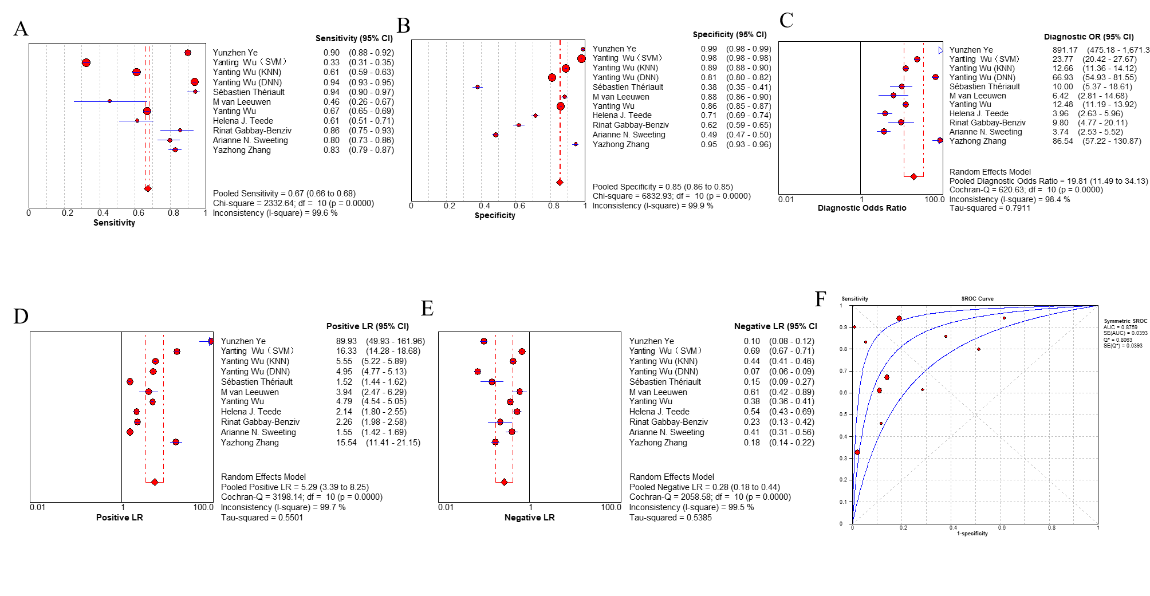
**

Fig.S.5. A-F. predict GDM with a history of GDM

**
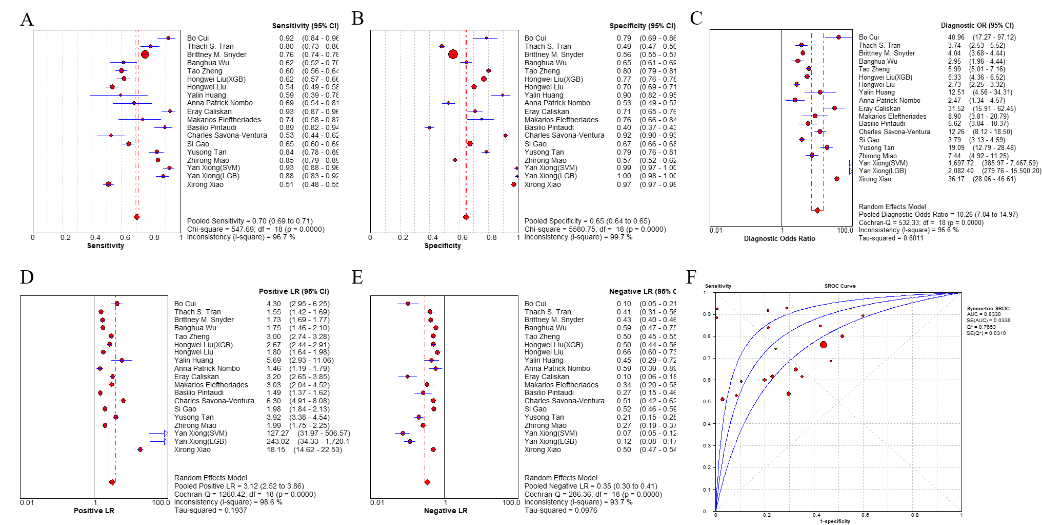
**

Fig.S.6. A-F. predict GDM without a history of GDM

**
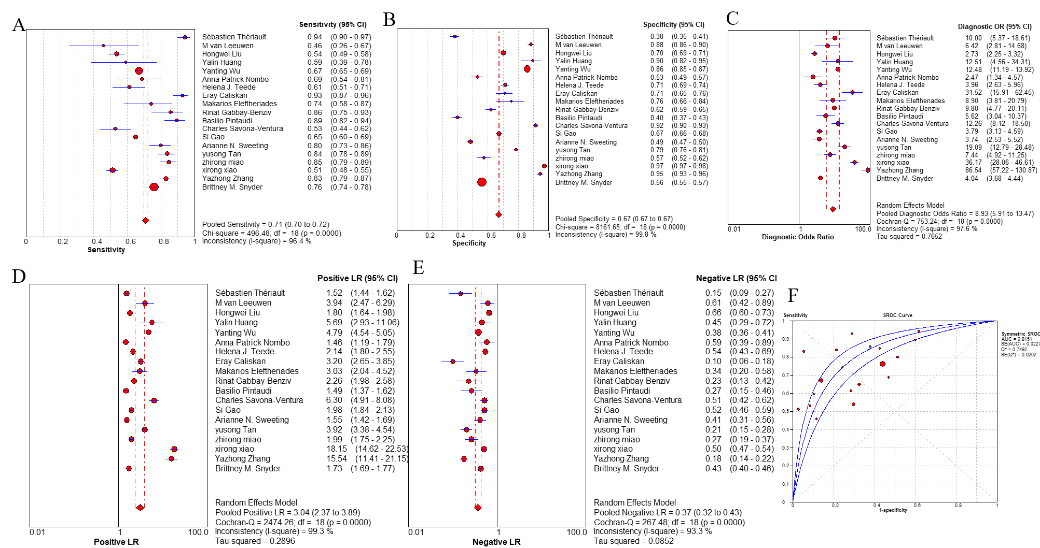
**

Fig.S.7. A-F. predict GDM with LR methods


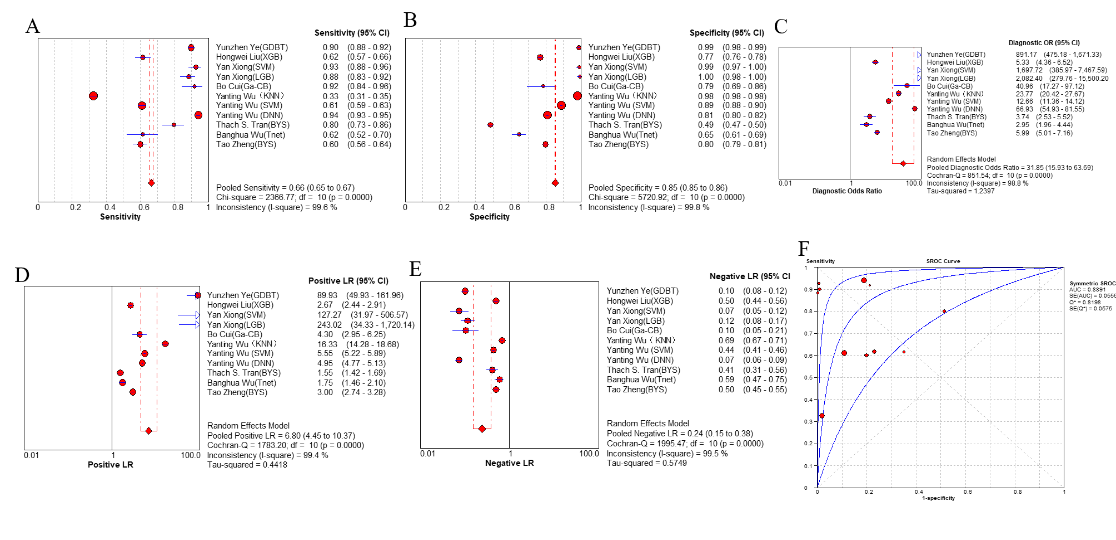


Fig.S.8. A-F. predict GDM with non-LR methods

**
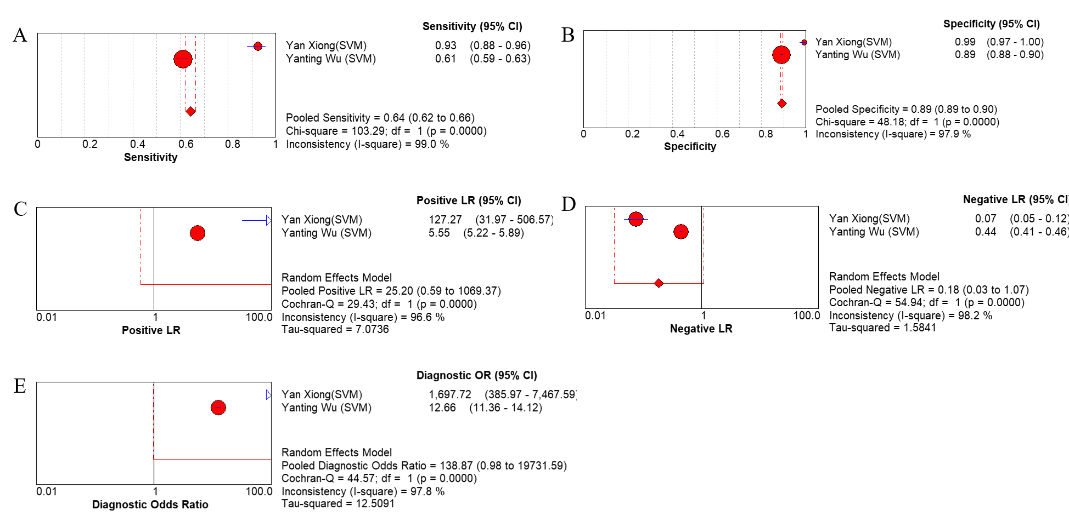
**

Fig.S.9. A-E. predict GDM with SVM methods

**
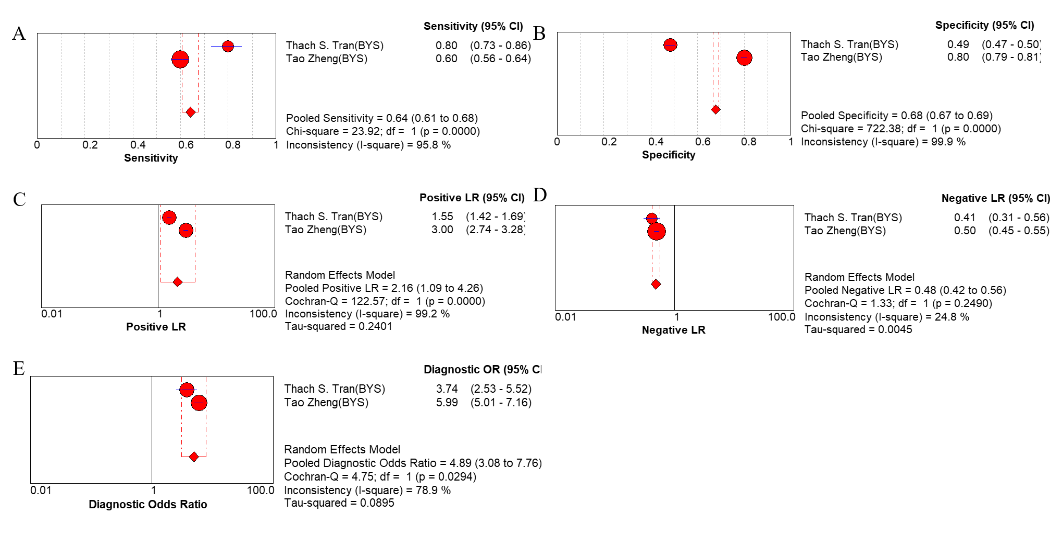
**

Fig.S.10. A-E. predict GDM with Bayesian methods

##### Table S8: Results of Meta-regression

When the DOR changes with diagnostic threshold, the ROC curve is asymmetrical. To study DOR variation in according to threshold, and thereby fit symmetrical or asymmetrical curves, the Moses-Shapiro-Littenberg method is used.

The method consists of studying this relationship by fitting the straight line$D=a+bS$

where D is the log of DOR and S a measure of threshold given by $S=In(\frac{Sen}{1-Sen}\times\frac{1-Spe}{Spe})$

Estimates of parameters a and b and their standard errors and covariance are obtained by ordinary or weighted least squares method using the NAG C library [9]. The weights can be simply the sample size or the inverse of variance of the log of the DOR.

The results of meta-regression showed that five items including sample size, country where the data from, the published year, ML methods, quality of models did not affect the diagnostic accuracy (p>0.05).

| Variety | Coeff. | Std. Err. | p – value | RDOR | [95%CI] |
| --- | --- | --- | --- | --- | --- |
| Cte. | 3.745 | 1.3390 | 0.0102 | ---- | ---- |
| S | -0.203 | 0.1797 | 0.2696 | ---- | ---- |
| SampleSize | -1.173 | 0.6556 | 0.0868 | 0.31 | (0.08;1.20) |
| Country | -0.878 | 1.0230 | 0.3994 | 0.42 | (0.05;3.45) |
| Year | -0.201 | 0.9329 | 0.8316 | 0.82 | (0.12;5.64) |
| MLmethod | 0.836 | 0.6784 | 0.2301 | 2.31 | (0.57;9.39) |
| Quality | -0.316 | 0.6033 | 0.6052 | 0.73 | (0.21;2.54) |

Tau-squared estimate = 2.1863 (Convergence is achieved after 7 iterations)

Restricted Maximum Likelihood estimation (REML)

| Variety | Coeff. | Std. Err. | p – value | RDOR | [95%CI] |
| --- | --- | --- | --- | --- | --- |
| Cte. | 3.505 | 0.7462 | 0.0001 | ---- | ---- |
| S | -0.214 | 0.1684 | 0.2164 | ---- | ---- |
| SampleSize | -1.145 | 0.6339 | 0.0834 | 0.32 | (0.09;1.18) |
| Country | -0.731 | 0.7453 | 0.3365 | 0.48 | (0.10;2.24) |
| MLmethod | 0.841 | 0.6626 | 0.2166 | 2.32 | (0.59;9.10) |
| Quality | -0.314 | 0.5907 | 0.5995 | 0.73 | (0.22;2.47) |

Tau-squared estimate = 2.0919 (Convergence is achieved after 6 iterations)

Restricted Maximum Likelihood estimation (REML)

| Variety | Coeff. | Std. Err. | p – value | RDOR | [95%CI] |
| --- | --- | --- | --- | --- | --- |
| Cte. | 3.371 | 0.6929 | 0.0001 | ---- | ---- |
| S | -0.221 | 0.1652 | 0.1939 | ---- | ----- |
| SampleSize | -1.101 | 0.6199 | 0.0879 | 0.33 | (0.09;1.19) |
| Country | -0.855 | 0.6963 | 0.2310 | 0.43 | (0.10;1.78) |
| MLmethod | 0.908 | 0.6385 | 0.1672 | 2.48 | (0.67;9.24) |

Tau-squared estimate = 2.0234 (Convergence is achieved after 7 iterations)

Restricted Maximum Likelihood estimation (REML)

| Var | Coeff. | Std. Err. | p - value | RDOR | [95%CI] |
| --- | --- | --- | --- | --- | --- |
| Cte. | 2.764 | 0.4892 | 0.0000 | ---- | ---- |
| S | -0.308 | 0.1507 | 0.0514 | ---- | ---- |
| SampleSize | -0.905 | 0.6038 | 0.1462 | 0.40 | (0.12;1.40) |
| MLmethod | 1.174 | 0.6073 | 0.0642 | 3.23 | (0.93;11.27) |

Tau-squared estimate = 2.0641 (Convergence is achieved after 6 iterations)

Restricted Maximum Likelihood estimation (REML)

| Var | Coeff. | Std. Err. | p - value | RDOR | [95%CI] |
| --- | --- | --- | --- | --- | --- |
| Cte. | 2.231 | 0.3395 | 0.0000 | ---- | ---- |
| S | -0.320 | 0.1522 | 0.0449 | ---- | ---- |
| MLmethod | 0.920 | 0.5894 | 0.1300 | 2.51 | (0.75;8.41) |

Tau-squared estimate = 2.1138 (Convergence is achieved after 7 iterations)

Restricted Maximum Likelihood estimation (REML)
