# Supplementary material for: Machine Learning Prediction Models for Gestational Diabetes Mellitus: Meta-analysis
Source: J Med Internet Res. 2022 Mar 16;24(3):e26634. doi: 10.2196/26634 (PMC8968560; doi:10.2196/26634)
Supplement: Multimedia Appendix 2 [file jmir_v24i3e26634_app2.docx]

Multimedia Appendix 2

(Machine Learning Prediction Models for Gestational Diabetes Mellitus: Meta-analysis)

##### Checklists from Prediction model Risk of Bias Assessment Tool (PROBAST)^13^.

| **DOMAIN 1: Participants** | | | |
| --- | --- | --- | --- |
| **A. Risk of Bias** | | | |
| *Describe the sources of data and criteria for participant selection:* | | | |
|  | | Dev | Val |
| - 1. Were appropriate data sources used, e.g. cohort, RCT or nested case-control study data? | |  |  |
| - 1. Were all inclusions and exclusions of participants appropriate? | |  |  |
| **Risk of bias introduced by selection of participants** | **RISK:**  *(low/ high/ unclear)* |  |  |
| *Rationale of bias rating:* | | | |
|  | | | |
| **B. Applicability** | | | |
| *Describe included participants, setting and dates:* | | | |
| **Concern that the included participants and setting do not match the review question** | **CONCERN:**  *(low/ high/ unclear)* |  |  |
| *Rationale of applicability rating:* | | | |
|  | | | |

| **DOMAIN 2: Predictors** | | | |
| --- | --- | --- | --- |
| **A. Risk of Bias** | | | |
| *List and describe predictors included in the final model, e.g. definition and timing of assessment:* | | | |
|  | | Dev | Val |
| - 1. Were predictors defined and assessed in a similar way for all participants? | |  |  |
| - 1. Were predictor assessments made without knowledge of outcome data? | |  |  |
| - 1. Are all predictors available at the time the model is intended to be used? | |  |  |
| **Risk of bias introduced by predictors or their assessment** | **RISK:**  *(low/ high/ unclear)* |  |  |
| *Rationale of bias rating:* | | | |
| **B. Applicability** | | | |
| Concern that the definition, assessment or timing of predictors in the model do not match the review question | **CONCERN:**  *(low/ high/ unclear)* |  |  |
| *Rationale of applicability rating:* | | | |

| **DOMAIN 3: Outcome** | | | |
| --- | --- | --- | --- |
| **A. Risk of Bias** | | | |
| *Describe the outcome, how it was defined and determined, and the time interval between predictor assessment and outcome determination:* | | | |
|  | | Dev | Val |
| - 1. Was the outcome determined appropriately? | |  |  |
| - 1. Was a pre-specified or standard outcome definition used? | |  |  |
| - 1. Were predictors excluded from the outcome definition? | |  |  |
| - 1. Was the outcome defined and determined in a similar way for all participants? | |  |  |
| - 1. Was the outcome determined without knowledge of predictor information? | |  |  |
| - 1. Was the time interval between predictor assessment and outcome determination appropriate? | |  |  |
| **Risk of bias introduced by the outcome or its determination** | **RISK:**  *(low/ high/ unclear)* |  |  |
| *Rationale of bias rating:* | | | |
| **B. Applicability** | | | |
| *At what time point was the outcome determined:*  *If a composite outcome was used, describe the relative frequency/distribution of each contributing outcome:* | | | |
| **Concern that the outcome, its definition, timing or determination do not match the review question** | **CONCERN:**  *(low/ high/ unclear)* |  |  |
| *Rationale of applicability rating:* | | | |

| **DOMAIN 4: Analysis** | | | |
| --- | --- | --- | --- |
| **Risk of Bias** | | | |
| *Describe numbers of participants, number of candidate predictors, outcome events and events per candidate predictor:* | | | |
| *Describe how the model was developed (for example in regards to modeling technique (e.g. survival or logistic modeling), predictor selection, and risk group definition):* | | | |
| *Describe whether and how the model was validated, either internally (e.g. bootstrapping, cross validation, random split sample) or externally (e.g. temporal validation, geographical validation, different setting, different type of participants):* | | | |
| *Describe the performance measures of the model, e.g. (re)calibration, discrimination, (re)classification, net benefit, and whether they were adjusted for optimism:* | | | |
| *Describe any participants who were excluded from the analysis:* | | | |
| *Describe missing data on predictors and outcomes as well as methods used for missing data:* | | | |
|  | | Dev | Val |
| - 1. Were there a reasonable number of participants with the outcome? i.e. Was the sample size adequately powered? | |  |  |
| - 1. Were continuous and categorical predictors handled appropriately? | |  |  |
| - 1. Were all enrolled participants included in the analysis? | |  |  |
| - 1. Were participants with missing data handled appropriately? | |  |  |
| - 1. Was selection of predictors based on univariable analysis avoided? | |  |  |
| - 1. Were complexities in the data accounted for appropriately? | |  |  |
| - 1. Were relevant model performance measures evaluated appropriately? | |  |  |
| - 1. Were model overfitting and optimism in model performance accounted for? | |  |  |
| - 1. Do predictors and their assigned weights in the final model correspond to the results from the reported multivariable analysis? | |  |  |
| **Risk of bias introduced by the analysis** | **RISK:**  *(low/ high/ unclear)* |  |  |
| *Rationale of bias rating:* | | | |

**Overall assessment**

| Use the following tables to reach overall judgements about risk of bias and concerns regarding applicability of the prediction model evaluation (development and/or validation) across all assessed domains.   \| **Reaching an overall judgement about risk of bias of the prediction model evaluation** \| \| \| --- \| --- \| \| **Low risk of bias** \| If all domains were rated low risk of bias.  If a prediction model was developed without any external validation, and it was rated as low risk of bias for all domains, consider downgrading to **high risk of bias**. Such a model can only be considered as low risk of bias, if the development was based on a very large data set and included some form of internal validation. \| \| **High risk of bias** \| If at least one domain is judged to be at **high risk of bias**. \| \| **Unclear risk of bias** \| If an unclear risk of bias was noted in at least one domain and it was low risk for all other domains. \|  \| **Reaching an overall judgement about applicability of the prediction model evaluation** \| \| \| --- \| --- \| \| **Low concerns regarding applicability** \| If low concerns regarding applicability for all domains, the prediction model evaluation is judged to have **low concerns regarding applicability**. \| \| **High concerns regarding applicability** \| If high concerns regarding applicability for at least one domain, the prediction model evaluation is judged to have **high concerns regarding applicability**. \| \| **Unclear concerns regarding applicability** \| If unclear concerns (but no “high concern”) regarding applicability for at least one domain, the prediction model evaluation is judged to have **unclear concerns regarding applicability** overall. \| |
| --- | --- | --- | --- | --- | --- | --- | --- | --- | --- | --- | --- | --- | --- | --- | --- | --- |

| **Overall judgement about risk of bias and applicability of the prediction model evaluation** | | |
| --- | --- | --- |
| **Overall judgement of risk of bias** | **RISK:**  *(low/ high/ unclear)* |  |
| *Summary of sources of potential bias:* | | |
| **Overall judgement of applicability** | **CONCERN:**  *(low/ high/ unclear)* |  |
| *Summary of applicability concerns:* | | |
